# Comparative Advantage of Agricultural Trade in Countries along the Belt and Road and China and Its Dynamic Evolution Characteristics

**DOI:** 10.3390/foods11213401

**Published:** 2022-10-28

**Authors:** Defeng Zhang, Zhilu Sun

**Affiliations:** Institute of Agricultural Economics and Development, Chinese Academy of Agricultural Sciences, Beijing 100081, China

**Keywords:** agricultural trade, comparative advantage, countries along the B&R, the B&R initiative, food security, agri-food systems

## Abstract

Trade is an important means to achieve the Sustainable Development Goals (SDGs) Target 2.1 “Zero Hunger”, and comparative advantage can be used to explain the causes and performance of trade. This study measures the static distribution of agricultural trade comparative advantage in countries along the Belt and Road (B&R) and China by utilizing the Balassa revealed comparative advantage (RCA) index, and further calculates its dynamic change by utilizing the revealed symmetric comparative advantage (RSCA) index and the ordinary least squares correlation analysis. The results show that: (1) in the face of multiple unfavorable factors, the initial comparative advantage of most agricultural products at Harmonized System (HS) 2-digit level in countries along the B&R and China deteriorated, simultaneously, but the initial comparative disadvantage of most and some agricultural products at HS 2-digit level in countries along the B&R and China improved, respectively; (2) the present agricultural trade comparative advantage in most countries along the B&R was higher than China and had a larger extent of change, but the current product structure of their bilateral agricultural trade was in line with each other’s comparative advantage, indirectly proving the validity of the Heckscher–Ohlin theorem. Our research findings suggest that the agricultural trade comparative advantage in countries along the B&R and China need to be further utilized to improve agricultural trade performance and better play its important role in ensuring global, regional, and national food security.

## 1. Introduction

The challenges to ending hunger, food insecurity, and all forms of malnutrition have kept growing in recent years [1], especially the ongoing COVID-19 pandemic outbreak in early 2020 and the ongoing war in Ukraine outbreak in February 2022, both of which have triggered new crises in global agri-food systems on top of existing challenges, such as climate change, economic slowdowns and food loss and waste that are already undermining the global community’s effort of achieving the Sustainable Development Goals (SDGs) Target 2.1 “Zero Hunger” adopted by all the United Nations (UN) member states in 2015 [2]. It is estimated that between 702 and 828 million people around the world were affected by hunger in 2021, which has grown by about 150 million since the outbreak of the COVID-19 pandemic [1].

Given that trade can move food from where it can be produced at a relatively low cost to where it is needed and contribute to building a better world free of hunger, the 2030 Agenda for Sustainable Development of the UN recognized trade as an important means to achieve the “Zero Hunger” target [3]. The global trade in food and agricultural products has more than doubled in volume and calories since 1995 [3]. While all economies have strengthened their participation in the global food and agricultural market, emerging and developing countries have become active participants and now account for about one-third of global food and agricultural trade [3,4]. However, in a time of unprecedented global changes and a once-in-a-century pandemic, the trends of trade protectionism and anti-globalization have gradually risen around the world in recent years, resulting in more frequent international trade frictions and the strengthening of local decoupling of global supply chain. Therefore, the instability and uncertainty of agricultural trade at global, regional, and national levels have increased significantly [2]. These challenges emphasize the need for more breakthrough research, especially a deeper understanding of the causes and performance of agricultural trade, and better approaches to facilitate international cooperation and promote a resilient agri-food system in every country [3].

In September and October of 2013, the Chinese government successively proposed the initiative of jointly building the Silk Road Economic Belt and the 21st Century Maritime Silk Road (hereinafter referred to as the B&R), respectively. Rooted in the principle of mutual consultation, joint efforts, and shared interests, the B&R Initiative is committed to building a free trade system and an open global economy, encouraging countries along the B&R to achieve coordination of economic policies and creating an inclusive regional economic cooperation architecture that benefits all involved stakeholders [5,6,7]. Promoting agricultural cooperation along the B&R, which is necessitated by the need for countries along the B&R and China to further expand and deepen the opening-up and for the world agriculture to grow in a sound and sustained way, by shaping the landscape of agricultural cooperation in the world and fostering orderly flow of factors, efficient allocation of resources and deep integration of markets along the B&R [5,6]. According to the data in the Food and Agriculture Organization Corporate Statistical Database (FAOSTAT) [8], in recent years, while exporting lots of grains, cotton, oilseeds, vegetable oil, sugar and meat, countries along the B&R were also importing lots of vegetables, fruits and aquatic preparations; and while importing lots of oilseeds, cereals, cotton, vegetable oil, sugar and animal products, China was also exporting lots of vegetables, fruits and aquatic preparations. Therefore, the agricultural trade between countries along the B&R and China has strong complementarity [9,10,11,12].

As a key principle in international trade and the basis of why free trade is beneficial to countries, the comparative advantage describes the tendency for a country to export a given commodity which is relatively more competitive throughout the rest of the world, and is widely used to evaluate patterns of trade performance [13,14]. The Heckscher– Ohlin theorem implies that, with free trade, a country will export the good that uses its relatively abundant factor intensively and import the good that uses its relatively scare factor intensively [15]. The validity of the Heckscher–Ohlin theorem can be tested by examining the comparative advantage [16]. Then, what are the evolution patterns of agricultural trade performance in countries along the B&R and China? Does the initial agricultural trade comparative advantage or disadvantage in countries along the B&R and China strengthen or weaken? Are there differences in agricultural trade comparative advantage between countries along the B&R and China? Does the agricultural trade between countries along the B&R and China validate the Heckscher–Ohlin theorem?

This study aims to provide a better understanding of the agricultural trade performance of countries along the B&R and China, and determine what actions should be taken to improve the agricultural trade performance to achieve more stable trade earnings. First, we present the current status of agricultural trade between countries along the B&R and China since the B&R Initiative was proposed in 2013. Second, by adopting the Balassa revealed comparative advantage (RCA) index, we measure the static distribution of agricultural trade comparative advantage in countries along the B&R and China from 2000 to 2018. Third, by using the revealed symmetric comparative advantage (RSCA) index and time series regression method, we calculate the dynamic change of the agricultural trade comparative advantage. Then, we put forward policy implications.

Our empirical results show that, in the face of multiple unfavorable factors, the initial comparative advantage of most agricultural products at a Harmonized System (HS) 2-digit level in countries along the B&R and China decreased, simultaneously, indicating that the agricultural trade performance in countries along the B&R and China tended to deteriorate. The present agricultural trade comparative advantage in most countries along the B&R was higher than China and had a larger extent of change, but the current product structure of their bilateral agricultural trade was in line with each other’s comparative advantage, indirectly proving the validity of the Heckscher–Ohlin theorem.

The remainder of this paper is organized as follows: Section 2 reviews the existing literature and clarifies the contributions of this paper. Section 3 provides the current situation of agricultural trade between countries along the B&R and China. Section 4 presents the materials and methods. Section 5 shows the results. Section 6 discusses the results. Section 7 summarizes the main conclusions of this paper, puts forward policy recommendations, and discusses the possible limitations of this paper and the future directions for research.

## 2. Literature Review

The concept of comparative advantage is a cornerstone of economic theory [17], and can be attributed to Ricardo’s theory of comparative advantage, generally known as the Ricardian theory [18], which put forward that different productive factors specialize in different economic activities based on their relative productivity differences. According to the Ricardian theory, differences in relative productivity determine the pattern of trade, and then the observable pattern of trade can be used to infer unobservable differences in relative productivity [19]. But many studies have pointed out the problems with the Ricardian theory, such as discontinuous adjustment, indeterminacy of final terms of trade and no income distribution effects [20,21,22]. By assuming countries have access to the same technology and share the same tastes, but differ in endowments of productive factors, Heckscher [23] and Ohlin [24] developed an analysis of trade on the differences in endowment, generally known as the Heckscher–Ohlin theorem, which put forward a different explanation of comparative advantage and is closer to the real world [25]. The Heckscher–Ohlin theorem predicts that countries produce relatively more of the goods that use their relatively abundant factors intensively, and asserts that differences in comparative advantage come from differences in factor abundance and factor intensity of goods [26]. The Heckscher–Ohlin theorem is generally acceptable for its explanatory role of national factor endowment differences in determining the pattern of trade according to comparative advantage [27], and based on which, a series of trade theories or approaches were proposed, such as the Heckscher–Ohlin–Samuelson theorem [28,29] and the Chamberlin–Heckscher–Ohlin approach [30].

With the development of the theory of comparative advantage, the evaluation of comparative advantage has attracted the attention of trade theorists. The comparative advantage can be broadly defined and usually measured by the RCA index, which was firstly proposed by Balassa [31]. Based on the economic efficiency of an industry, the Balassa RCA index is easily calculated and widely used, and can reveal a country’s weak and strong export sectors and provide a simple way to evaluate a country’s trade policy [13,14]. Thereafter, based on the Balassa RCA index, a series of improved RCA indexes have been proposed, including the revealed competitiveness (RC) index [32], the relative trade advantage (RTA) index [33], the RSCA index [34,35], the weighted RCA index [36], the additive RCA index [37], the normalized RCA index [38], the “regression-based” index [39], the modified RCA index [40], the contribution to trade balance (CTB) index [41], and the new class of RCA indexes [15]. Computed on the basis of trade data, these RCA indexes can provide synthetic measures of comparative advantage [42].

There are several ways of using the RCA indexes in measuring trade performance, including comparing the calculated value with the neutral point, comparing given sectors by using the calculated value, and comparing the calculated value at the national or regional level [14,43]. Given that agricultural trade is an important part of overall economic activity, continues to play a major role in domestic agricultural production and employment and also plays a fundamentally important role in global food security, some existing studies measured the comparative advantage of agricultural trade at national and regional levels by utilizing the RCA indexes, such as Serbia [40], Hungary [44], Russia [45], China [46], Pakistan [47], Indonesia [48], Myanmar [14], India [49], Brazil [50], Canada [51], and Ghana [52]. Despite the utilization of the RCA indexes analysis over the past decades, the existing research on the comparative advantage of countries along the B&R and China mainly focuses on the manufacturing industry, and there were relatively few studies on the comparative advantage of agricultural trade in the two sides from a dynamic perspective [35,53,54,55].

Despite their contributions, the existing studies on agricultural trade comparative advantage in countries along the B&R and China are still insufficient. First, with regard to the measures, most of the existing studies only used the static indexes such as the RCA index and the modified RCA index, resulting in the inability to reveal the dynamic change of agricultural trade comparative advantage, and whether the comparative advantage has a solidification or liquidity change trend in a certain period. Second, in terms of trade data, most of the existing studies used an incomplete classification and definition of traded agricultural products at the HS 2-digit level—with HS01–HS24 and without HS50 (silk), HS51 (wool, fine or coarse animal hair, etc.), HS52 (cotton), and HS53 (vegetable textile fibers, etc.), but the latter 4 categories are important agricultural products with large trade volume both in countries along the B&R and China, resulting in the trade data analyzed in existing studies being smaller than the actual scale.

This paper attempts to quantitatively evaluate and comparatively analyze the static distribution and dynamic change of comparative advantage of agricultural trade in countries along the B&R and China, and qualitatively analyze the unfavorable factors undermining the agricultural trade comparative advantage in countries along the B&R and China. This study contributes to the existing research in two ways. First, this paper is the first to employ the RSCA index and the time series regression method to calculate the dynamic change of agricultural trade comparative advantage in countries along the B&R and China, and provide new evidence about the evolution patterns of agricultural trade performance in countries along the B&R and China. Second, in accordance with the 1999 HS of Commodity Coding at 2-digit level, we define traded agricultural products with reference to World Trade Organization (WTO)’s “Agricultural Agreement”, specifically covering HS01–HS24, HS50 (HS5001–HS5003), HS51 (HS5101–HS5105), HS52 (HS5201–HS5203) and HS53 (HS5301–HS5305), and totally including 28 varieties of agricultural products at HS 2-digit level in this study, which fully depicts the real situation of agricultural trade in countries along the B&R and China.

## 3. The Current Situation of Agricultural Trade between Countries along the B&R and China

### 3.1. The Evolutionary Trends of Agricultural Trade between Countries along the B&R and China

According to Table 1, from 2013 to 2018, the total volume of agricultural trade between countries along the B&R and China increased from USD 40.48 billion to USD 51.03 billion with a cumulative growth rate of 26.1%, which means that, since the B&R initiative was proposed, the agricultural trade between the two sides achieved rapid development. The proportion of agricultural trade between countries along the B&R and China in China’s agricultural trade increased from 21.88% in 2013 to 23.57% in 2018. China’s agricultural exports to countries along the B&R continued to rise with an average annual growth rate of 5.57%, and its proportion in China’s agricultural exports was higher than 30% since 2015. China’s agricultural imports from countries along the B&R declined in 2015 and 2016, but its proportion in China’s agricultural imports remained between 18% and 20% during 2013–2018. All these highlight the important role of countries along the B&R in China’s agricultural trade. From the perspective of China’s trade balance, agricultural trade between countries along the B&R and China presented a surplus in 2016 and 2017 and a trade deficit in the rest years.

### 3.2. The Product Structure of Agricultural Trade between Countries along the B&R and China

According to Table 2, in 2018, HS07 (edible vegetables and certain roots and tubers), HS08 (edible fruits and nuts, etc.), HS03 (fish and crustaceans, etc.), HS20 (preparations of vegetables, fruits, nuts, etc.), HS16 (meat, fish, crustaceans, mollusks, etc.), HS21 (miscellaneous edible preparations), HS12 (oilseeds and oleaginous fruits, etc.), HS09 (coffee, tea, mate and spices), HS17 (sugars and sugars confectionery), and HS05 (animal originated products, etc.) were the top 10 categories of agricultural products that China exported to countries along the B&R. In total agricultural exports, in 2018 compared with 2013, the export proportion of HS07 (edible vegetables and certain roots and tubers), HS08 (edible fruits and nuts, etc.), HS21 (miscellaneous edible preparations), HS12 (oilseeds and oleaginous fruits, etc.), HS09 (coffee, tea, mate and spices), HS17 (sugars and sugars confectionery) and HS05 (animal originated products, etc.) increased, while that of HS03 (fish and crustaceans, etc.), HS20 (preparations of vegetables, fruits, nuts, etc.), and HS16 (meat, fish, crustaceans, mollusks, etc.) decreased. The CR (concentration ratio) 5 and CR10 of China’s agricultural exports to countries along the B&R were 64.9% and 84.3% in 2013, respectively, and then were 60.8% and 83.0% in 2018, respectively.

In 2018, HS15 (animal, vegetable or microbial fats and oils, etc.), HS08 (edible fruits and nuts, etc.), HS03 (fish and crustaceans, etc.), HS10 (cereals), HS07 (edible vegetables and certain roots and tubers), HS11 (products of the milling industry, etc.), HS12 (oilseeds and oleaginous fruits, etc.), HS16 (meat, fish, crustaceans, mollusks, etc.), HS23 (food industries, residues and wastes, etc.), and HS19 (preparations of cereals, flour, starch or milk, etc.) were the top 10 categories of agricultural products that China imported from countries along the B&R. In total agricultural imports, in 2018 compared with 2013, the import proportion of HS08 (edible fruits and nuts, etc.), HS03 (aquatic products), HS10 (cereals), HS11 (products of the milling industry, etc.), HS12 (oilseeds and oleaginous fruits, etc.), HS16 (meat, fish, crustaceans, mollusks, etc.), and HS23 (food industries, residues and wastes, etc.) increased, while that of HS15 (animal, vegetable or microbial fats and oils, etc.), HS07 (edible vegetables and certain roots and tubers), and HS19 (preparations of cereals, flour, starch or milk, etc.) decreased. The CR5 and CR10 of China’s agricultural import from countries along the B&R were 75.1% and 90.4% in 2013, respectively, and then were 67.0% and 82.8% in 2018, respectively.

Therefore, since the B&R initiative was proposed, both of the product concentration of agricultural products exported from China to countries along the B&R and imported from countries along the B&R to China tended to decline, and both of the product structure of agricultural export and import became more diversified. There were significant differences and complements in main exported and imported agricultural products between countries along the B&R and China.

### 3.3. The Market Structure of Agricultural Trade between Countries along the B&R and China

According to Table 3, in 2018, Vietnam, Thailand, Malaysia, Indonesia, the Philippines, Russia, Singapore, Myanmar, India, and U.A.E (United Arab Emirates) were the top 10 agricultural export destinations along the B&R for China. In total agricultural imports, in 2018 compared with 2013, the import proportion of Vietnam, Indonesia, the Philippines, and Myanmar increased, while that of Thailand, Malaysia, Russia, Singapore, India, and the U.A.E. decreased. The CR5 and CR10 of China’s agricultural exports to countries along the B&R were 60.1% and 79.7% in 2013, respectively, and then were 62.9% and 80.6% in 2018, respectively.

In 2018, Thailand, Indonesia, Vietnam, Russia, Malaysia, India, Ukraine, the Philippines, Pakistan, and Mongolia were the top 10 agricultural import origins along the B&R for China. In total agricultural exports, in 2018 compared with 2013, the export proportion of Thailand, Indonesia, Vietnam, Russia, Ukraine, the Philippines, Pakistan, and Mongolia increased, while that of Malaysia and India decreased. The CR5 and CR10 of China’s agricultural import from countries along the B&R were 74.9% and 92.1% in 2013, respectively, and 72.7% and 90.1% in 2018, respectively.

Therefore, since the B&R initiative was proposed, the market concentration of agricultural products exported from China to countries along the B&R tended to rise, while that of agricultural products imported from China to countries along the B&R tended to decrease, and both of export destinations and import origins along the B&R for China became more concentrated. In recent years, most Southeast Asian countries, Russia and India were the main agricultural export destinations and import origins along the B&R for China simultaneously.

## 4. Materials and Methods

### 4.1. Methods

Different measures of comparative advantage are appropriate for different purposes [16], and each of the RCA indexes has pros and cons, thus it is important to understand the properties of the indexes well and properly use them [14]. The Balassa RCA index is the most widely used measure for revealing the static distribution of comparative advantage, and is also the basis of most of the other RCA indexes. Initially, the Balassa RCA index reveals a comparative advantage if its value is greater than 1 or a comparative disadvantage if its value is less than 1 [31]. The two intervals do not have the same length, one is finite while the other is left-bounded only, resulting in the asymmetric issue that the measurement of comparative advantage or disadvantage is not on the same numerical basis [35]. The RSCA index proposed by Dalum et al. [34], based on the Balassa RCA index, can effectively deal with the asymmetric issue. Therefore, in this study, to measure the static distribution of the agricultural trade comparative advantage in countries along the B&R and China, the Balassa RCA index is employed, and then, to evaluate the dynamic change of the agricultural trade comparative advantage, the RSCA index and the ordinary least squares correlation analysis proposed by Kocourek [56] based on the RSCA index are used.

#### 4.1.1. The Balassa RCA Index

The Balassa RCA index posits that patterns of trade among countries are governed by their relative differences in productivity as well as relative costs and non-price factors [31]. Although such productivity differences are difficult to observe, a Balassa RCA metric can be readily calculated using trade data to reveal such differences. The Balassa RCA index measures comparative advantage in exporting certain commodities to partner country under the context of free trade, and shows comparative advantage or disadvantage in exporting certain commodities [41,57]. The Balassa RCA index is defined as the ratio of a country’s total exports of the commodity to its total exports divided by share of world exports of the same commodity in total world export [31], and can be defined as:

(1) $$RCA_{i,t}^{A}=\frac{X_{i,t}^{A}}{\sum_{i\in P}X_{t}^{A}}/\frac{X_{i,t}^{W}}{\sum_{i\in P}X_{t}^{W}}$$

where $RCA_{i,t}^{A}$ represents country A’s RCA value of a given agricultural product *i* in year *t*; $X_{i,t}^{A}$ and $X_{i,t}^{W}$ denote the exports of agricultural product *i* of country A and the world in year *t*, respectively; $\sum_{i\in P}X_{t}^{A}$ and $\sum_{i\in P}X_{t}^{W}$ denote the exports of total agricultural product of country A and the world in year *t*, respectively; A is countries along the B&R or China; W is the world; and *i* is a particular class of agricultural products, and *P* is the set of all agricultural products (with i∈P).

The Balassa RCA index takes a value between 0 and +∞. Originally, 1 was considered to be the only neutral value of the index, resulting in the asymmetric issue. Recently, more subdivided value ranges of the index have been set to deal with the asymmetric issue in some literature, such as Rodas-Martini [16] and Long [46]. With reference to Rodas-Martini [16] and Long [46], the following value classification of the Balassa RCA index is employed in this study. $RCA_{i,t}^{A}\in [0,\;0.80)$ is supposed to reveal a comparative disadvantage, $RCA_{i,t}^{A}\in [0.80,\;1.25)$ is supposed to reveal a medium comparative advantage, $RCA_{i,t}^{A}\in [1.25,\;2.50)$ is supposed to reveal a strong comparative advantage, and $RCA_{i,t}^{A}\in [2.50,+\infty )$ is supposed to reveal a very strong comparative advantage. Country A with comparative advantage in product *i* is considered to have a relative export strength in that product, and the higher the value of $RCA_{i,t}^{A}$, the stronger its comparative advantage in product *i*.

#### 4.1.2. The RSCA Index

Since the Balassa RCA index results in an output which cannot be compared on both sides of a reasonable neutral value, the RSCA index was proposed by Dalum et al. [34] and Laursen [35] to solve the asymmetric issue of the Balassa RCA index, which is likely to violate the assumption of normality of the error term in regression analysis and to produce unreliable *t*-statistics. The RSCA index can be defined as:

(2) $$RSCA_{i,t}^{A}=\frac{RCA_{i,t}^{A}-1}{RCA_{i,t}^{A}+1}$$

where $RSCA_{i,t}^{A}$ represents country A’s RSCA value of a given agricultural product *i* in year *t*. The RSCA index ranges from −1.0 to +1.0 with 0 as the neutral value. The closer to +1.0 the value of $RSCA_{i,t}^{A}$ becomes, the stronger the comparative advantage of agricultural product *i* in year *t* in country A, while the greater the values of $RSCA_{i,t}^{A}$ that are converging to −1.0, the more substantial is the comparative disadvantage of agricultural product *i* in year *t* in country A.

The RSCA index is also a measure of relative not absolute strength, which implies that, regardless of how poorly or strongly a country is performing, by definition, the country will be specialized in something, and will always have high values of RSCA for some sectors and low values for other sectors [35]. The RSCA index has the advantage of attributing changes below 0 the same weight as changes above 0, and is the best of the alternatives discussed with respect to normality [34].

#### 4.1.3. The Ordinary Least Squares Correlation Analysis

In order to measure the mean annual change pace of comparative advantage and reveal the magnitude and direction of the change, Kocourek proposed the ordinary least squares correlation analysis [56]. Specifically, taking the RSCA value in a given sample period and the year as the dependent variable and independent variable, respectively, a time series regression is conducted. The slope in the regression is the average change range of comparative advantage, and can be defined as:

(3) $$\beta_{i,t}^{A}=\frac{n\sum_{t\in n}RSCA_{i,t}^{A}t-\sum_{t\in n}RSCA_{i,t}^{A}\sum_{t\in n}t}{n\sum_{t\in n}t^{2}-{\left(\sum_{t\in n}t\right)}^{2}}$$

where $\beta_{i,t}^{A}$ represents the estimated annual paces of change of $RSCA_{i,t}^{A}$; *i* denotes a given agricultural product; *t* denotes a period of time; and *n* denotes the number of years. The values of $\beta_{i,t}^{A}$ are tested for statistical significance utilizing *t*-test, and only the values of $\beta_{i,t}^{A}$ at 95% statistical significance are accepted [56].

### 4.2. Definition of Countries along the B&R

Referring to the Statistical Bulletin of China’s Outward Direct Investment, jointly issued by the Ministry of Commerce of China, the National Bureau of Statistics of China and the State Administration of Foreign Exchange of China [58], countries along the B&R include 64 countries in this study, specifically consisting of 11 countries in Southeast Asia (Indonesia, Malaysia, the Philippines, Singapore, Thailand, Brunei, Vietnam, Laos, Myanmar, Cambodia, and Timor-Leste), 6 countries in Central Asia and Northeast Asia (Kazakhstan, Kyrgyzstan, Tajikistan, Turkmenistan, Uzbekistan, and Mongolia), 8 countries in South Asia (India, Pakistan, Sri Lanka, Bangladesh, Nepal, Bhutan, Maldives, and Afghanistan), 16 countries in West Asia and North Africa (Iran, Saudi Arabia, Iraq, Turkey, Syria, Jordan, Israel, Palestine, Bahrain, Qatar, Yemen, Oman, United Arab Emirates, Kuwait, Lebanon, and Egypt), and 23 countries in Central and Eastern Europe and Southern Europe (Hungary, Czech Republic, Slovak Republic, Poland, Russia, Ukraine, Belarus, Moldova, Georgia, Armenia, Azerbaijan, Estonia, Latvia, Lithuania, Albania, Serbia, Bosnia and Herzegovina, Bulgaria, Romania, Croatia, Macedonia, Montenegro, and Slovenia).

### 4.3. Data Source

In this study, agricultural products include 28 varieties, and are further merged into 4 categories as shown in Table 4. The data of agricultural trade used in this study is retrieved from the United Nations Commodity Trade Statistics Database (UN COMTRADE) (https://comtrade.un.org/data/ (accessed on 16 May 2022)). Based on the availability and completeness of the data, the sample period in the rest of this study is determined to be 2000–2018.

## 5. Results

### 5.1. The Static Distribution of Agricultural Trade Comparative Advantage in Countries along the B&R and China

#### 5.1.1. The Distribution of Agricultural Trade Comparative Advantage in Countries along the B&R

**Figure 1:**
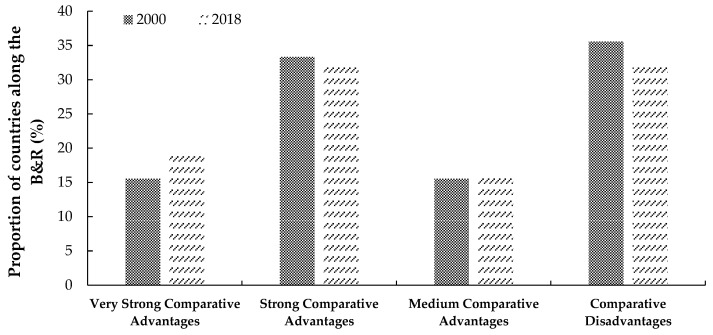
The distribution of comparative advantage of total agricultural trade in countries along the B&R in 2000 and 2018 by using the Balassa RCA index. The *x*-axis represents different levels of comparative advantage; and the *y*-axis represents the proportion of number of countries along the B&R with different levels of comparative advantage in 64 countries along the B&R.

Based on Equation (1), we measured the comparative advantage of total agricultural trade in each country along the B&R in each year from 2000 to 2018, and then, according to the value classification proposed by Rodas-Martini [16] and Long [44], we drew the distribution of agricultural trade comparative advantage in 2000 and 2018, as shown in Figure 1. From 2000 to 2018, in 64 countries along the B&R, the total proportion of number of countries along the B&R with very strong, strong and medium comparative advantage increased from 64.4% to 68.0%, of which that with a very strong comparative advantage increased from 15.6% to 19.6%, while that with a comparative disadvantage decreased from 35.6% to 32.0%. Therefore, compared with the world average level, the comparative advantage of total agricultural trade in countries along the B&R totally strengthened during 2000–2018.

#### 5.1.2. The Position of China’s Agricultural Trade Comparative Advantage in Countries along the B&R

**Figure 2:**
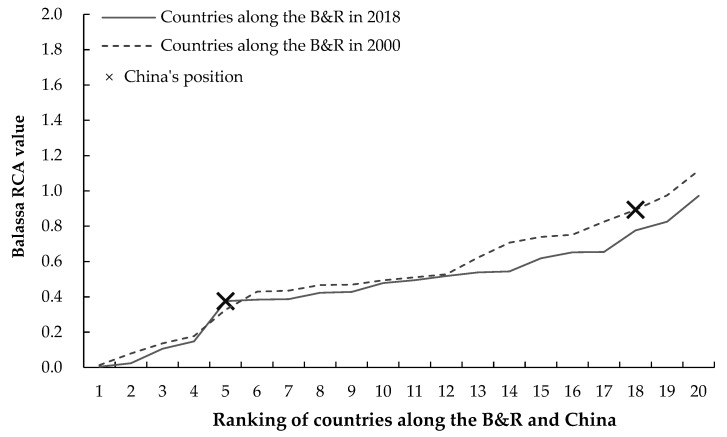
The Balassa RCA values of total agricultural trade in countries along the B&R and China in 2000 and 2018. The *x*-axis represents the ranking of countries along the B&R and China based on the Balassa RCA values in 2000 and 2018; and the *y*-axis represents the Balassa RCA value.

In order to clarify the position of China’s agricultural trade comparative advantage in countries along the B&R, the Balassa RCA values of agricultural trade in countries along the B&R and China in 2000 and 2018 were compared at the level of total agricultural products and 4 categories of agricultural products, respectively. Figure 2 presents the Balassa RCA values of total agricultural trade in China and countries along the B&R in 2000 and 2018. In 2000, the Balassa RCA value of China’s total agricultural trade was 0.89, supposing to reveal a medium comparative advantage and ranking 28th in countries along the B&R; and in 2018, the Balassa RCA value of China’s total agricultural trade fell to 0.37, supposing to reveal a comparative disadvantage and only ranking 52nd in countries along the B&R. Therefore, the comparative advantage of China’s total agricultural trade has significantly deteriorated from 2000 to 2018, degenerating from a medium comparative advantage to a comparative disadvantage, and the ranking of which in countries along the B&R has significantly decreased accordingly.

**Figure 3:**
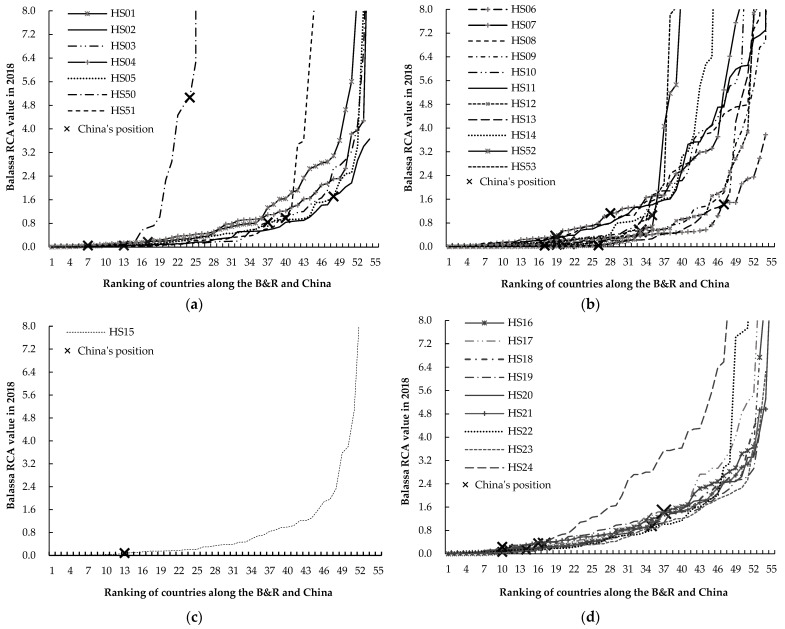
The Balassa RCA values of 4 categories of agricultural products in countries along the B&R and China in 2018. (**a**)the first category of agricultural products (live animals and animal products, including HS01–HS05 and HS50–HS51); (**b**) the second category of agricultural products (plant products, including HS06–HS14 and HS52–HS53); (**c**) the third category of agricultural products (animal and vegetable oils and fats, including HS15); (**d**) the fourth category of agricultural products (food, beverages and tobacco, including HS16–HS24). The *x*-axis represents the ranking of countries along the B&R and China based on the Balassa RCA values in 2018; and the *y*-axis represents the Balassa RCA value.

Subsequently, we analyzed the Balassa RCA values of 4 categories of agricultural products in countries along the B&R and China, respectively. According to Figure 3a, with regard to the first category of agricultural products (live animals and animal products), for countries along the B&R in 2018, the comparative advantage of HS01 (live animals), HS04 (dairy products, bird’s eggs, etc.) and HS50 (silk) was significantly higher than the other 4 varieties of agricultural products; and more than 25% countries had strong or very strong comparative advantage, such as HS01 (live animals) in Myanmar, HS04 (dairy products, bird’s eggs, etc.) in Belarus and HS50 (silk) in Vietnam. For China in 2018, the Balassa RCA values of HS05 (animal originated products, etc.) and HS50 (silk) ranked 6th and 3rd in countries along the B&R, respectively, with the former having a strong comparative advantage and the latter having a very strong comparative advantage; and that of HS02 (meat and edible meat offal) and HS04 (dairy products, bird’s eggs, etc.) ranked 42nd and 48th in countries along the B&R, respectively, both with a significant comparative disadvantage.

According to Figure 3b, with regard to the second category of agricultural products (plant products), for countries along the B&R in 2018, the comparative advantage of HS07 (edible vegetables and certain roots and tubers), HS08 (edible fruits and nuts, etc.), HS10 (cereals), and HS11 (products of the milling industry, etc.) was significantly higher than the other 7 varieties of agricultural products; more than 40% countries had a strong or very strong comparative advantage, such as HS07 (edible vegetables and certain roots and tubers) in Turkey, HS08 (edible fruits and nuts, etc.) in Vietnam, HS10 (cereals) in Thailand and Vietnam, and HS11 (products of the milling industry, etc.) in Kazakhstan; and more than 80% countries had a comparative disadvantage in HS06 (live trees and other plants, etc.) and HS13 (lac, gums, etc.). For China in 2018, the Balassa RCA values of HS13 (lac, gums, etc.) and HS14 (vegetable planting materials, etc.) ranked 7th and 13th in countries along the B&R, respectively, with the former having a strong comparative advantage and the latter having a medium strong comparative advantage.

According to Figure 3c, with regard to the third category of agricultural products (animal and vegetable oils and fats), for countries along the B&R in 2018, a strong or very strong comparative advantage of HS15 (animal, vegetable or microbial fats and oils, etc.) existed in only 12 countries, such as Indonesia and Ukraine. For China in 2018, HS15 (animal, vegetable or microbial fats and oils, etc.) has significant comparative disadvantage, only ranking 44th in countries along the B&R.

According to Figure 3d, with regard to the fourth category of agricultural products (food, beverages and tobacco), for countries along the B&R in 2018, more than 25% countries had a strong or very strong comparative advantage, for example, 28 countries had a strong or very strong comparative advantage in HS24 (tobacco and manufactured tobacco substitutes, etc.) such as Indonesia, and 21 countries had a strong or very strong comparative advantage in HS17 (sugar and sugar confectionery) such as Pakistan and Myanmar. For China in 2018, the Balassa RCA values of HS16 (meat, fish, crustaceans, mollusks, etc.) and HS20 (preparations of vegetables, fruits, nuts, etc.) ranked 19th and 21th in countries along the B&R, specifically, with the former having strong comparative advantage and the latter having medium strong comparative advantage; and that of HS15 (animal and vegetable oils and fats, etc.), HS18 (cocoa and its products), and HS24 (tobacco and its products) ranked 44th, 43rd, and 43rd in countries along the B&R, respectively, all with significant comparative disadvantage.

### 5.2. The Dynamic Change in Agricultural Trade Comparative Advantage in Countries along the B&R and China

#### 5.2.1. The Distribution of the RSCA Values of Agricultural Trade in Countries along the B&R

**Figure 4:**
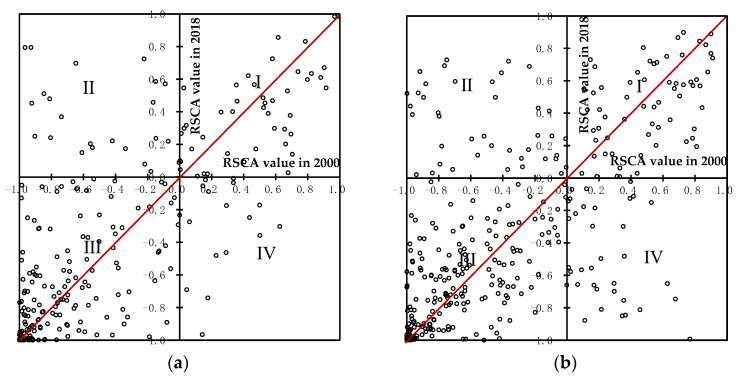

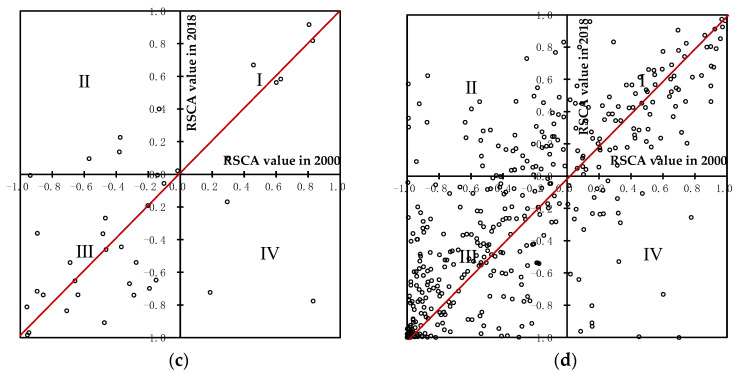
The distribution of the RSCA values of 4 categories of agricultural products in countries along the B&R in 2000 and 2018. (**a**) the first category of agricultural products (live animals and animal products, including HS01–HS05 and HS50–HS51); (**b**) the second category of agricultural products (plant products, including HS06–HS14 and HS52–HS53); (**c**) the third category of agricultural products (animal and vegetable oils and fats, including HS15); (**d**) the fourth category of agricultural products (food, beverages and tobacco, including HS16–HS24). The *x*-axis represents the RSCA value in 2000; and the *y*-axis represents the RSCA value in 2018.

Based on the measurement results of 4 categories of agricultural products in countries along the B&R in 2000 and 2018 by using the RSCA index, the changes in the RSCA values were presented in Figure 4. The *x*-axis and *y*-axis represent the zero values of the RSCA index in 2018 and 2000, respectively, and divide the coordinate series into 4 quadrants, reflecting 4 different combinations of the RSCA values in 2000 and 2018. Points in the quadrant I indicate the maintenance of the initial comparative advantage, points in the quadrant II indicate the acquisition of a new comparative advantage, points in the quadrant III indicate the maintenance of the initial comparative disadvantage, and points in the quadrant IV indicate the deterioration of the initial comparative advantage.

According to Figure 4a–d, for countries along the B&R, we found that: (1) 4 categories of agricultural products were mainly distributed in the quadrant III, especially the first and second categories, including between 63.0% and 64.9% of countries, which indicated that the first and second categories in most countries along the B&R had comparative disadvantage in 2000 and still maintain it in 2018, indicating a strong solidification in the comparative disadvantage. (2) 4 categories of agricultural products had the relatively least distribution in quadrant IV, especially the second category, including 9% of countries as shown in Figure 4b, showing that most agricultural products in countries along the B&R had maintained their initial comparative advantage, and the loss of comparative advantage was also mainly concentrated in the second category. (3) The relatively largest distribution in quadrants I and II was the fourth category as shown in Figure 4d, which indicated that the fourth category in countries along the B&R not only maintained its initial comparative advantage, but also acquired new comparative advantage, making its comparative advantage stronger.

#### 5.2.2. The Mean Annual Change Pace of Agricultural Trade Comparative Advantage in Countries along the B&R and China

**Figure 5:**
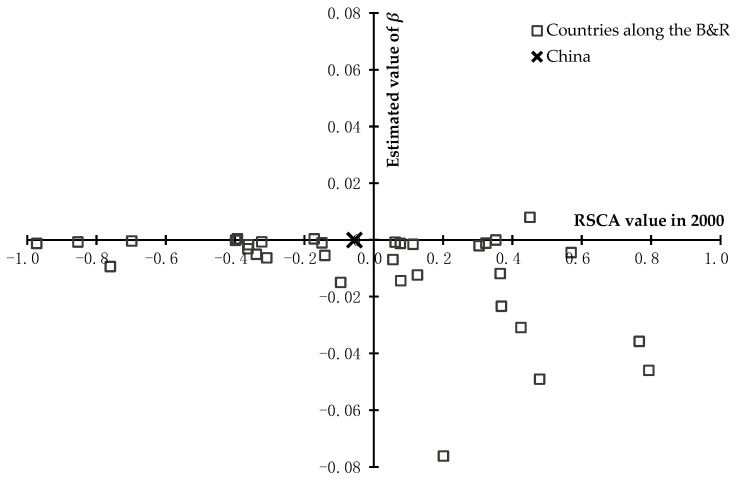
The mean annual change pace of the RSCA values of total agricultural products in countries along the B&R and China from 2000 to 2018. The *x*-axis represents the RSCA value in 2000; and the *y*-axis represents the estimated value of $\beta_{i,t}^{A}$. $\beta_{i,t}^{A}>0$ is supposed to reveal an increase in comparative advantage from 2000 to 2018, and $\beta_{i,t}^{A}<0$ is supposed to reveal a decrease in comparative advantage from 2000 to 2018.

**Figure 6:**
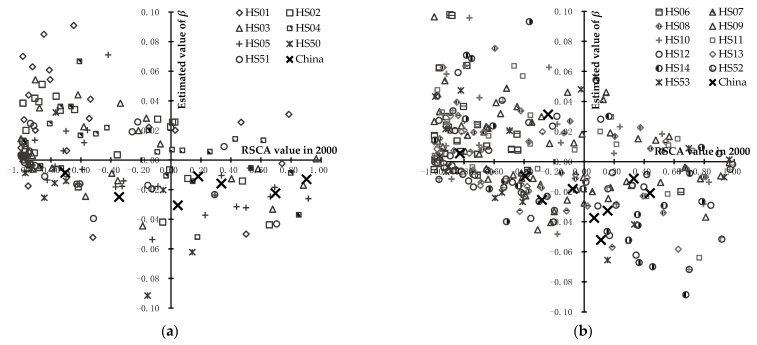

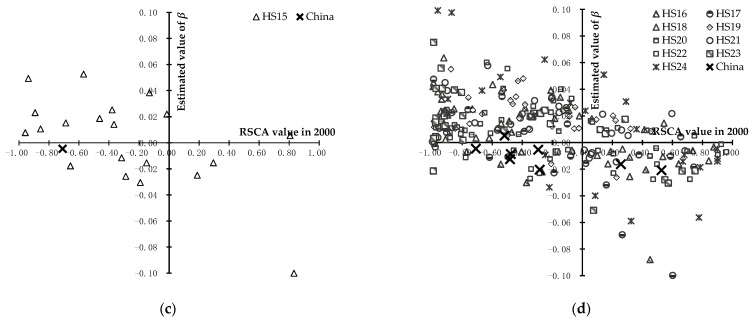
The mean annual change pace of the RSCA values of 4 categories of agricultural products in countries along the B&R and China from 2000 to 2018. (**a**) the first category of agricultural products (live animals and animal products, including HS01–HS05 and HS50–HS51); (**b**) the second category of agricultural products (plant products, including HS06–HS14 and HS52–HS53); (**c**) the third category of agricultural products (animal and vegetable oils and fats, including HS15); (**d**) the fourth category of agricultural products (food, beverages and tobacco, including HS16–HS24). The *x*-axis represents the RSCA value in 2000; and the *y*-axis represents the estimated value of $\beta_{i,t}^{A}$. $\beta_{i,t}^{A}>0$ is supposed to reveal an increase in comparative advantage from 2000 to 2018, and $\beta_{i,t}^{A}<0$ is supposed to reveal a decrease in comparative advantage from 2000 to 2018.

Based on Equation (3), the mean annual change pace of comparative advantage of total agricultural products and 4 categories of agricultural products in countries along the B&R and China from 2000 to 2018 were calculated, and then shown in Figure 5 and Figure 6, respectively. According to Figure 5, with regard to total agricultural products, for countries along the B&R, the change in agricultural trade comparative advantage was positive for Iran and Philippines, but negative for other countries; and for countries with RSCA>0 in 2000, denoting an initial comparative advantage, the decrease in comparative advantage was greater, but for country with RSCA<0 in 2000, denoting an initial comparative disadvantage, the change in comparative disadvantage was smaller. For China, the change in agricultural trade comparative advantage was negative, but the extent of the change was smaller than most countries along the B&R.

According to Figure 6a, with regard to the first category of agricultural products (live animals and animal products), for countries along the B&R, the initial comparative advantage of HS01 (live animals) and HS02 (meat and edible meat offal) increased in most countries, for example, that of HS01 (live animals) increased in 21 countries such as Thailand and Kyrgyzstan; and, in total, agricultural products at the HS 2-digit level had a significant increase in initial comparative disadvantage, of which HS05 (animal originated products, etc.) was the top 1 category of agricultural products in the number of countries, specifically including 18 countries such as Vietnam and Singapore. For China, all the initial comparative advantage of seven varieties of agricultural products decreased, of which HS01 (live animals) had the largest decline. Therefore, the initial comparative advantage totally improved for most agricultural products at the HS 2-digit level in most countries along the B&R, but deteriorated for all agricultural products at the HS 2-digit level in China, and the extent of the change was greater in most countries along the B&R.

According to Figure 6b, with regard to the second category of agricultural products (plant products), for countries along the B&R, the initial comparative advantage of HS06 (live trees and other plants, etc.) and HS13 (lac, gums, etc.) increased in most countries, for example, that of HS06 (live trees and other plants, etc.) increased in 20 countries such as Iran and Belarus, and in total, agricultural products at an HS 2-digit level had a greater increase for lower initial comparative advantage and a greater decrease for higher initial comparative advantage; and HS12 (oilseeds and oleaginous fruits, etc.) and HS14 (vegetable planting materials, etc.) were the top 2 categories of agricultural products in number of countries with a decrease in initial comparative advantage, for example, HS12 (oilseeds and oleaginous fruits, etc.) in 19 countries such as Vietnam and Kyrgyzstan. For China, except for HS06 (live trees and other plants, etc.) and HS13 (lac, gums, etc.) with initial comparative disadvantage, all the other agricultural products at the HS 2-digit level presented a downward trend in initial comparative advantage, especially HS10 (cereal). Therefore, in countries along the B&R and China, the initial comparative advantage of agricultural products at the HS 2-digit level deteriorated, the initial comparative disadvantage of which improved, and the extent of the change was smaller in China.

According to Figure 6c, with regard to the third category of agricultural products (animal and vegetable oils and fats), for countries along the B&R, the initial comparative advantage of HS15 (animal, vegetable or microbial fats and oils, etc.) increased in 12 countries such as Russia and Belarus, but decreased in eight countries such as Singapore and Vietnam. For China, the initial comparative disadvantage of HS15 (animal, vegetable or microbial fats and oils, etc.) deteriorated further, but the extent of the change was smaller than most countries along the B&R.

According to Figure 6d, with regard to the fourth category of agricultural products (food, beverages and tobacco), for countries along the B&R, the initial comparative advantage of HS24 (tobacco and its products) decreased in most countries; and the initial comparative disadvantage of HS19 (preparations of cereals, flour, starch or milk, etc.), HS21 (miscellaneous edible preparations), and HS23 (food industries, residues and wastes, etc.) improved in most countries, for example, that of HS23 (food industries, residues and wastes, etc.) in 23 countries such as Indonesia and Singapore. For China, except for HS17 (sugars and sugars confectionery) with an initial comparative disadvantage, the initial comparative advantage of the rest agricultural products at HS 2-digit level decreased, but the extent of the change was larger in most countries along the B&R.

## 6. Discussion

This study revisited the topic of comparative advantage of agricultural trade in countries along the B&R and China from the perspective of static distribution and dynamic change, which expanded existing studies on agricultural trade in countries along the B&R and China and provided new empirical evidence on comparative advantage of agricultural trade. We found that the second and fourth categories of agricultural products had stronger comparative advantage than the first and third categories in countries along the B&R, and there were significant national and varieties differences in agricultural trade comparative advantage. For example, most countries in Central and Eastern Europe are endowed with abundant resources for developing agriculture accounting for 15–20% of gross domestic product (GDP), such as large amounts of fertile farmland, and have comparative advantage in land-intensive agricultural products, such as HS04 (dairy products, birds’ eggs, natural honey, etc.), HS10 (cereals), and HS19 (preparations of cereals, flour, starch or milk, etc.) [59,60,61,62]. Most countries in Southeast Asia have a tropical humid climate, and have comparative advantage in water-intensive agricultural products, such as HS08 (edible fruits and nuts, etc.), HS10 (cereals), HS16 (meat, fish, crustaceans, mollusks, etc.) and HS17 (sugars and sugar confectionery) [63,64]. For China, the comparative advantage of total agricultural trade ranked low position in countries along the B&R; and in 28 varieties of agricultural products, only the comparative advantage of HS05 (other animal products), HS16 (meat, fish, crustaceans, mollusks, etc.), and HS50 (silk) ranked high position in countries along the B&R, all of which are labor-intensive or processed agricultural products.

Therefore, the comparative advantage of agricultural trade in countries along the B&R and China had significant varieties differences, and in total, that in countries along the B&R was stronger than China, which is consistent with previous studies [46,53,54,55]. The comparative advantage of most agricultural products at the HS 2-digit level in countries along the B&R and China deteriorated, simultaneously, while the comparative disadvantage of some agricultural products at the HS 2-digit level in countries along the B&R improved. The main agricultural products exported respectively by countries along the B&R and China were just the agricultural products with comparative advantage, which verified that the product structure of agricultural trade between the two sides was in line with each other’s comparative advantage and had strong complementary, indirectly proving the validity of the Heckscher–Ohlin theorem.

It should be noted that currently, by affecting the orderly operation of industrial chain and supply chain in the agricultural sector, some unfavorable factors have undermined the agricultural trade comparative advantage in countries along the B&R and China, and further weakened the resilience of their food security. For countries along the B&R, the mainly unfavorable factors are as follows:

(1) The agricultural factor markets in many countries along the B&R are not perfect, restricting the improvement of agricultural production capacity and the scale of tradable agricultural products. Taking the agricultural land as an example, given that well-functioning agricultural land markets are a precondition for agricultural and rural development, the agricultural land markets remain weak and face many constraints including informalities, technical errors, and complicated and costly land transaction procedures in many countries along the B&R, such as countries in Eastern Europe and Central Asia [61,65].
(2) The agriculture in many countries is increasingly shifting to intensive and market-oriented structure, reducing the sustainability and versatility of agriculture. Typically following market demands and economic opportunities, subsistence agricultural products are being replaced with cash agricultural products in many countries along the B&R, which improves overall income for smallholders, but often occurs at the expense of ecological and environmental sustainability, as well as livelihood security, especially in Southeast Asian countries [66,67,68].
(3) The logistics infrastructure of agriculture in many countries along the B&R is relatively backward, affecting the sustained and stable growth of agricultural trade with China. Most countries along the B&R are developing countries and generally lack adequate funding and technology to promote the interconnection of infrastructure to the outside world [69,70], resulting in insufficient international logistics support capabilities and hindering the circulation of production factors and commodities with China. According to Table 5, among the 141 economies with data in 2019 retrieved from The Global Competitiveness Report 2019 [71], the transportation infrastructure conditions in most of the main countries along the B&R ranked below 50th, and were also significantly lower than China.
(4) The degree of trade facilitation in countries along the B&R is generally low, resulting in relatively high agricultural trade costs with China. The WTO defines trade costs incurred in the process of cross-border trade as transportation costs, policy barriers, information and transaction costs, contract execution costs and regulatory costs, and cumbersome customs and port clearance procedures, shortage of trade infrastructure, frequent changes in laws and regulations, high information costs, lack of property rights protection, and weak contract execution efficiency will lead to a significant increase in trade costs [72]. According to Table 6, among the 190 economies with data in 2020, the facilitation degree of trading cross borders in most of the main countries along the B&R ranked below 60th, and the time and cost of imports and exports of most of the main countries along the B&R were significantly higher than China, regardless of border compliance or document compliance.
(5) The COVID-19 epidemic and regional conflicts have weakened the resilience of agricultural supply chains in countries along the B&R. The former has caused severe disruption of agricultural supply chains—including restrictions on labor and interruption of transport, processing, retailing and input distribution, and highlighted the fragilities in regional and global agri-food systems, especially in countries along the B&R [73,74,75]. The latter especially the war in Ukraine have led to a decline in the scale of regional and global agricultural trade, deeply affecting the ability of some vulnerable food-importing countries to meet their needs and the ability of international agencies to provide food aid to countries that are suffer from famine, by the rising risks of disruptions to regional and global agricultural market and agricultural trade policy interventions [1,76,77,78].

**Table 5:** The traffic infrastructure conditions in main countries along the B&R and China in 2019.

Countries	Ranking of Transport Infrastructure in 141 Economies	Road		Railroad		Shipping and Seaport	
		Road Connectivity (0–100)	Quality of Road Infrastructure (1–7)	Railroad Density (km/1000 km^2^)	Efficiency of Train Services (1–7)	Liner Shipping Connectivity (0–100)	Efficiency of Seaport Services (1–7)
China	24	95.7	4.6	7.2	4.5	100.0	4.5
Poland	25	88.0	4.3	60.5	3.9	63.1	4.5
India	28	75.8	4.5	22.7	4.4	59.9	4.5
Turkey	33	87.1	5.0	13.3	3.5	59.7	4.7
Saudi Arabia	34	100.0	5.2	0.7	4.5	66.6	4.8
Serbia	46	84.5	3.5	42.7	2.6	—	3.1
Russia	49	85.7	3.5	5.2	4.9	40.4	4.7
Thailand	53	80.0	4.4	8.7	2.8	48.0	4.1
Indonesia	55	59.8	4.2	2.6	4.7	47.8	4.3
Ukraine	59	78.2	3.0	37.3	4.2	30.1	3.9
Romania	61	79.3	3.0	46.8	2.8	29.8	3.9
Vietnam	66	63.3	3.4	7.6	3.6	68.8	3.8
Pakistan	69	80.2	4.0	10.1	3.8	38.2	4.1
Kazakhstan	73	79.3	3.6	5.9	4.2	—	3.3
Philippines	102	51.6	3.7	1.7	2.4	29.0	3.7
Mongolia	119	59.2	3.1	1.2	3.5	—	1.6

Notes: road connectivity measures average speed and straightness of a driving itinerary connecting the 10 or more largest cities that together account for at least 15% of the economy’s total population, and the scale of which ranges from 0 to 100 (excellent); quality of road infrastructure is measured based on the survey question “In your country, what is the quality (extensiveness and condition) of road infrastructure?”, and 1 denotes extremely poor—among the worst in the world and 7 denotes extremely good—among the best in the world; railroad density is measured by kilometers of railroad per 1000 square km of land; efficiency of train services is measured based on the survey question “In your country, how efficient (i.e., frequency, punctuality, speed, price) are train transport services?”, and 1 denotes extremely inefficient—among the worst in the world and 7 denotes extremely efficient—among the best in the world; liner shipping connectivity is scored on the Liner Shipping Connectivity Index, which assesses a country’s connectivity to global shipping networks, and the scale of which ranges from 0 to 100 (excellent); efficiency of seaport services is measured based on the survey question “In your country, how efficient (i.e., frequency, punctuality, speed, price) are seaport services?”, and 1 denotes extremely inefficient—among the worst in the world and 7 denotes extremely efficient—among the best in the world; and “—“ denotes the data is unavailable.

**Table 6:** The facilitation degree of trading cross borders in main countries along the B&R and China in 2020.

Countries	Ranking of Trading across Borders in 190 Economies	Export				Import			
		Border Compliance Time (Hours)	Border Compliance Cost (USD)	Documentary Compliance Time (Hours)	Documentary Compliance Cost (USD)	Border Compliance Time (Hours)	Border Compliance Cost (USD)	Documentary Compliance Time (Hours)	Documentary Compliance Cost (USD)
Poland	1	0.0	0.0	0.5	0.0	0.0	0.0	0.5	0.0
Romania	1	0.4	0.0	0.5	0.0	0.4	0.0	0.5	0.0
Serbia	23	4.1	47.3	2.3	35.0	4.6	52.0	3.0	35.0
Turkey	44	9.8	338.0	4.0	55.0	6.5	46.0	2.0	55.0
China	56	20.7	256.2	8.6	73.6	35.7	241.3	12.8	77.3
Thailand	62	44.0	222.6	11.3	96.9	50.2	232.5	4.0	43.5
India	68	52.1	211.9	11.6	58.0	65.3	266.1	19.9	100.0
Ukraine	75	6.0	75.0	66.0	192.0	32.0	100.0	48.0	162.0
Saudi Arabia	86	37.0	319.0	11.0	73.0	72.0	464.5	32.0	267.0
Russia	99	66.0	580.0	25.4	92.0	30.0	520.0	42.5	152.5
Vietnam	104	55.0	290.0	50.0	139.2	56.0	373.0	76.0	182.5
Kazakhstan	105	105.0	470.0	128.0	200.0	2.0	0.0	5.5	0.0
Pakistan	111	58.0	288.0	55.0	118.0	120.0	287.0	96.0	130.0
Philippines	113	42.5	456.0	36.0	52.5	120.0	689.5	96.0	67.5
Indonesia	116	56.3	211.1	61.3	138.8	99.4	382.6	106.2	164.4
Mongolia	143	134.0	225.1	168.0	63.9	48.0	209.8	114.7	82.6

Notes: ranking of economies on the ease of trading across borders is determined by sorting their scores for trading across borders; time for border compliance to export or import records the time associated with compliance with the economy’s customs regulations and with regulations relating to other inspections that are mandatory in order for the export or import shipment to cross the economy’s border, as well as the time and cost for handling that takes place at its port or border; cost for border compliance to export or import records the cost associated with compliance with the economy’s customs regulations and with regulations relating to other inspections that are mandatory in order for the export or import shipment to cross the economy’s border, as well as the time and cost for handling that takes place at its port or border; time for documentary compliance to export or import records the time associated with compliance with the export or import documentary requirements of all government agencies of the origin economy, the destination economy and any transit economies; and cost for documentary compliance to export or import records the cost associated with compliance with the export or import documentary requirements of all government agencies of the origin economy, the destination economy and any transit economies. The data were retrieved from the historical data of the Doing Business Legacy (https://www.worldbank.org/en/programs/business-enabling-environment/doing-business-legacy (accessed on 16 May 2022)).

Since the accession to the WTO in December 2001, the Chinese government has consistently and significantly opened up domestic agricultural markets in accordance with its commitments, by eliminating preferential policies for agricultural export, reducing agricultural subsidies and cutting agricultural tariffs, which has also made domestic agricultural producers especially smallholders directly face fierce international competition [46,54,79,80,81]. However, the protection degree of international agricultural markets especially the invisible protection in many developed countries is still very high, not only the scale of agricultural subsidies showing a trend of increasing, but also the measures of sanitary and phytosanitary (SPS) and technical barriers to trade (TBT) showing a trend of continuous improvement of standards and more complex procedures in recent years [3,82]. With limited land resources and an aging rural labor force, the prices of input factors in agricultural production such as labor, land, and chemical fertilizers have continued to rise in China in recent years, resulting in the continuous growth of agricultural production costs and the significant decrease in international competitiveness and comparative advantage of agricultural trade [53,83,84,85].

## 7. Conclusions and Policy Implications

### 7.1. Conclusions

This study quantitatively measures the static distribution of agricultural trade comparative advantage in countries along the B&R and China by utilizing the Balassa RCA index, and then calculates the dynamic change of agricultural trade comparative advantage by utilizing the RSCA index and the ordinary least squares correlation analysis. We draw the following conclusions. First, the initial comparative advantage of most agricultural products at HS 2-digit level in countries along the B&R and China decreased, simultaneously, but the initial comparative disadvantage of most and some agricultural products at HS 2-digit level in countries along the B&R and China improved, respectively. Second, the present agricultural trade comparative advantage in most countries along the B&R was at a higher position with a faster rate of change than China, but the current product structure of their bilateral agricultural trade was in line with each other’s comparative advantage, indirectly proving the validity of the Heckscher–Ohlin theorem. Third, by affecting the operation of industrial chain and supply chain in the agricultural sector, some unfavorable factors undermined the agricultural trade comparative advantage in countries along the B&R and China, and further weakened the resilience of their food security.

### 7.2. Policy Implications

Considering that the comparative advantage of most traded agricultural products at HS 2-digit level in countries along the B&R and China deteriorated from 2000 to 2018, there is still big room for the improvement in agricultural trade patterns and policies of the two sides, and in this way can the performance of their agricultural trade be further improved and the role of agricultural trade in ensuring food security be better played. The policy implications of this paper are as follows. First, the agricultural export portfolio between countries along the B&R and China needs to be further improved based on their comparative advantage, and the orderly flow of agricultural elements, the efficient allocation of agricultural resources, the deep integration of regional agricultural markets, and the comprehensive coordination of national development initiatives or strategies need to be promoted, such as the B&R Initiative of China, the Vision 2030 of Saudi Arabia, the Eastern Economic Corridor of Thailand, and the “Bright Road” Initiative of Kazakhstan. Second, the knowledge sharing, technology transfer, information communication, and personnel exchanges in the agricultural field of countries along the B&R and China need to be strengthened. Third, the genuine multilateralism needs to be adhered to together by countries along the B&R and China, including giving full play to the important role of the FAO, the International Fund for Agricultural Development (IFAD) and the World Food Programme (WFP) in global food security governance, and taking coordinated action to deal with the challenges of regional and global agricultural trade, such as climate change, the COVID-19 epidemic and regional conflicts.

### 7.3. Limitations and Suggestions for Future Research

This study has a few limitations. First, this paper tried to reveal the overall characteristics of agricultural trade comparative advantage in countries along the B&R, but considering that countries along the B&R are widely distributed and have significant differences in agricultural resource endowments, technology and policies, the characteristics of each country may not be consistent with the overall characteristics. Second, the RCA indexes employed in this paper do not fully take the distortions in international trade into account, such as the tariff and non-tariff restrictions, subsidies, trade agreements and exchange rate misalignments [86], resulting in the measurement results possibly being biased and their external validity in other countries remains to be verified. Third, owing to the unavailability of latest data, this paper only qualitatively analyzed the possible impact of the COVID-19 pandemic and the war in Ukraine on agricultural trade comparative advantage in countries along the B&R.

Future research directions need to be addressed. First, with the continuous improvement of the measures of comparative advantage, the evaluation of agricultural trade comparative advantage in countries along the B&R and China should also be adjusted accordingly, especially in each country along the B&R, so that the real status can be better revealed, and then more scientific evidence for the improvement of agricultural trade performance can be provided. Second, the COVID-19 pandemic and the war in Ukraine are still ongoing, their impact on agricultural trade comparative advantage in countries along the B&R and China and beyond needs to be constantly observed and quantitatively assessed.

## Figures and Tables

**Table 1:** The agricultural trade between countries along the B&R and China and its proportion in China’s agricultural trade during 2013–2018.

Year	China’s Exports		China’s Imports		China’s Exports and Imports		China’s Trade Balance
	To Countries along the B&R (Billion US$)	Proportion in China’s Agricultural Exports (%)	From Countries along the B&R (Billion US$)	Proportion in China’s Agricultural Imports (%)	Between China and Countries along the B&R (Billion US$)	Proportion in China’s Agricultural Trade (%)	Between China and Countries along the B&R (Billion US$)
2013	18.46	27.51	22.38	18.98	40.48	21.88	–3.92
2014	20.35	28.52	22.79	18.76	43.14	22.37	–2.44
2015	21.07	30.02	22.52	19.43	43.59	23.42	–1.45
2016	22.28	30.68	20.47	18.51	42.75	23.33	1.81
2017	22.92	30.50	22.53	18.07	45.45	22.75	0.39
2018	24.21	30.52	26.82	19.56	51.03	23.57	–2.61

Note: China’s trade balance equals to the difference between China’s agricultural exports to countries along the B&R and China’s agricultural imports from countries along the B&R.

**Table 2:** The top 10 categories of agricultural products at Harmonized System (HS) 2-digit level in their proportion in total agricultural trade between countries along the B&R and China in 2013 and 2018.

Ranking	China’s Exports to Countries along the B&R				China’s Imports from Countries along the B&R			
	2013		2018		2013		2018	
	Products at HS 2-Digit Level	Proportion in Total Exports (%)	Products at HS 2-Digit Level	Proportion in Total Exports (%)	Products at HS 2-Digit Level	Proportion in Total Imports (%)	Products at HS 2-Digit Level	Proportion in Total Imports (%)
1	HS07	18.4	HS07	20.2	HS15	31.8	HS15	23.9
2	HS08	16.6	HS08	16.8	HS52	13.8	HS08	15.3
3	HS03	11.9	HS03	9.9	HS08	10.6	HS03	13.5
4	HS20	9.9	HS20	7.9	HS03	9.7	HS10	9.4
5	HS16	8.1	HS16	6.0	HS07	9.2	HS07	4.9
6	HS21	4.4	HS21	5.5	HS10	5.1	HS11	4.0
7	HS12	4.2	HS12	4.6	HS11	3.2	HS12	3.4
8	HS17	3.9	HS09	4.4	HS19	2.8	HS16	3.2
9	HS09	3.5	HS17	4.1	HS23	2.1	HS23	2.9
10	HS24	3.4	HS05	3.6	HS12	2.1	HS19	2.3
Trade concentration	CR5	64.9	CR5	60.8	CR5	75.1	CR5	67.0
	CR10	84.3	CR10	83.0	CR10	90.4	CR10	82.8

Note: HS denotes Harmonized System classification; CR5 represents the cumulative proportion of exports and imports of the top 5 categories of agricultural products in total agricultural exports and imports, respectively; and CR10 represents the cumulative proportion of exports and imports of the top 10 categories of agricultural products in total agricultural exports and imports, respectively.

**Table 3:** The top 10 countries along the B&R in agricultural trade and their proportion in total agricultural trade amount with China in 2013 and 2018.

Ranking	China’s Exports to Countries along the B&R				China’s Imports from Countries along the B&R			
	2013		2018		2013		2018	
	Countries	Proportion in Total Exports (%)	Countries	Proportion in Total Exports (%)	Countries	Proportion in Total Imports (%)	Countries	Proportion in Total Imports (%)
1	Malaysia	14.1	Vietnam	21.8	Thailand	18.6	Thailand	20.8
2	Thailand	13.8	Thailand	13.6	Malaysia	17.2	Indonesia	19.2
3	Vietnam	12.6	Malaysia	10.0	Indonesia	15.4	Vietnam	12.1
4	Russia	10.7	Indonesia	9.0	India	14.6	Russia	11.9
5	Indonesia	8.9	Philippines	8.5	Vietnam	9.1	Malaysia	8.7
6	Philippines	7.6	Russia	8.1	Russia	7.0	India	5.5
7	Singapore	4.5	Singapore	3.5	Uzbekistan	2.6	Ukraine	5.1
8	India	3.1	Myanmar	2.2	Ukraine	3.4	Philippines	3.7
9	U.A.E.	2.8	India	2.0	Philippines	2.3	Pakistan	1.8
10	Poland	1.6	U.A.E	1.9	Singapore	1.9	Mongolia	1.3
Trade concentration	CR5	60.1	CR5	62.9	CR5	74.9	CR5	72.7
	CR10	79.7	CR10	80.6	CR10	92.1	CR10	90.1

Note: U.A.E. denotes United Arab Emirates; CR5 represents the cumulative proportion of exports and imports of the top 5 countries in total agricultural exports and imports, respectively; and CR10 represents the cumulative proportion of exports and imports of the top 10 countries in total agricultural exports and imports, respectively.

**Table 4:** The 4 categories of agricultural products based on the 28 varieties of agricultural products at HS 2-digit level.

Category	Name of Category
First category (including HS01–HS05 and HS50–HS51)	Live animals and animal products
Second category (including HS06–HS14 and HS52–HS53)	Plant products
Third category (including HS15)	Animal and vegetable oils and fats
Fourth category (including HS16–HS24)	Food, beverages and tobacco

## Data Availability

The data presented in this study are available on request from the corresponding author.
